# Neurobiological foundations of neurologic music therapy: rhythmic entrainment and the motor system

**DOI:** 10.3389/fpsyg.2014.01185

**Published:** 2015-02-18

**Authors:** Michael H. Thaut, Gerald C. McIntosh, Volker Hoemberg

**Affiliations:** ^1^Center for Biomedical Research in Music, Colorado State UniversityFort Collins, CO, USA; ^2^Department of Neurology, University of Colorado HealthFort Collins, CO, USA; ^3^SRH Neurorehabilitation Hospital Bad WimpfenBad Wimpfen, Germany

**Keywords:** auditory rhythm, entrainment, music, neurologic music therapy, rehabilitation

## Abstract

Entrainment is defined by a temporal locking process in which one system’s motion or signal frequency entrains the frequency of another system. This process is a universal phenomenon that can be observed in physical (e.g., pendulum clocks) and biological systems (e.g., fire flies). However, entrainment can also be observed between human sensory and motor systems. The function of rhythmic entrainment in rehabilitative training and learning was established for the first time by Thaut and colleagues in several research studies in the early 1990s. It was shown that the inherent periodicity of auditory rhythmic patterns could entrain movement patterns in patients with movement disorders (see for a review: Thaut et al., 1999). Physiological, kinematic, and behavioral movement analysis showed very quickly that entrainment cues not only changed the timing of movement but also improved spatial and force parameters. Mathematical models have shown that anticipatory rhythmic templates as critical time constraints can result in the complete specification of the dynamics of a movement over the entire movement cycle, thereby optimizing motor planning and execution. Furthermore, temporal rhythmic entrainment has been successfully extended into applications in cognitive rehabilitation and speech and language rehabilitation, and thus become one of the major neurological mechanisms linking music and rhythm to brain rehabilitation. These findings provided a scientific basis for the development of neurologic music therapy.

## PRINCIPLES OF ENTRAINMENT

In 1666, the Dutch physicist Christian Huygens discovered that the pendulum frequencies of two clocks mounted on the same wall or board became synchronized to each other. He surmised that the vibrations of air molecules would transmit small amounts of energy from one pendulum to the other and synchronize them to a common frequency. However, when set on different surfaces the effect disappeared. The transmitting medium was actually the vibrating board or wall. For air molecule vibrations there would have been too much dampening in the process of energy transmission, as was later discovered. The effect was subsequently confirmed by many other experiments and was called entrainment. In entrainment the different amounts of energy transferred between the moving bodies due to the asynchronous movement periods cause negative feedback. This feedback drives an adjustment process in which the different energy amounts are gradually eliminated to zero until both moving bodies move in resonant frequency or synchrony. The stronger “oscillator” locks the weaker into its frequency. When both oscillating bodies have equally strong energy, both systems move toward each other: the faster system slows down and the slower system speeds up until they lock into a common movement period (Pantaleone, 2002).

Technically, entrainment in physics refers to the frequency locking of two oscillating bodies, i.e., bodies that can move in stable periodic or rhythmic cycles. They have different frequencies or movement periods when moving independently, but when interacting they assume a common period. Incidentally, Huygens’ pendulums actually assumed a common period 180°out of phase, which he called “odd sympathy.” It is now known that entrainment can occur in various phase relationships of the movement onsets of the oscillating bodies. A stable phase relationship is achieved when both bodies start and stop their movement period at the same time. However, this is not a necessary prerequisite for entrainment to occur. The deciding factor for entrainment is the common period of the oscillating movements of the two bodies. The common period entrainment is of critical importance for clinical applications of rhythmic entrainment as a temporal cue in motor rehabilitation (Kugler and Turvey, 1987; Thaut et al., 1998a). Common period entrainment establishes that the rhythmic cue provides a continuous time reference during the complete duration of the movement to be regulated.

## AUDITORY SYSTEM AND TIME PERCEPTION

The importance of the auditory system in the control of movement was traditionally given much lesser attention in motor control theory and research than the visual or proprioceptive system. Therefore, auditory rhythm and more complex auditory time structures associated with musical patterns were not give much functional value in motor learning or motor rehabilitation. Consequently, application to motor therapies played no role in traditional music therapy. Music was assigned mostly a motivational role for movement performance (Thaut, 2005).

However, basic neurophysiology and biophysics of sensorimotor connectivity have always shown intriguing interactions between the auditory and the motor system. The ability of the auditory system to rapidly construct stable temporal templates is well known (see for a review: Thaut and Kenyon, 2003). The auditory system is superbly constructed to detect temporal patterns in auditory signals with extreme precision and speed, as required by the nature of sound as only existing in temporal vibration patterns (Moore, 2003). In those tasks, the auditory system is faster and more precise than the visual and tactile systems (Shelton and Kumar, 2010). Since sound waves that are most important for speech and music and other perceptual tasks are based on periodic motions that repeat themselves in regularly recurring cycles, the auditory system is also perceptually geared toward detecting and constructing rhythmic sound patterns. Finally, many studies have now shown that auditory rhythmic cues can entrain motor responses. For example, Thaut et al. (1998b) demonstrated that finger and arm movements instantaneously entrain to the period of a rhythmic stimulus (e.g., metronome beat) and stay locked to the metronome frequency even when subtle tempo changes are induced into the metronome that are consciously not perceived. These findings have been confirmed by other studies (cf, Large et al., 2002).

## NEURAL ENTRAINMENT

The neural basis for auditory-motor entrainment is lesser understood. Two early electrophysiological studies (Paltsev and Elner, 1967; Rossignol and Melvill Jones, 1976) showed how sound signals and rhythmic music can prime and time muscle activation via reticulospinal pathways. It is now well-established that the auditory system has richly distributed fiber connections to motor centers from the spinal cord upward on brain stem, subcortical, and cortical levels (Koziol and Budding, 2009; Schmahmann and Pandya, 2009; Felix et al., 2011). Although the specific basis of neural entrainment mechanisms remains not fully explored, several studies have at least been able to link neural oscillation patterns in the auditory system to the time and frequency dynamics of rhythmic sound stimuli. Fujioka et al. (2012) showed modulations in neuromagnetic beta oscillations related to the rhythmic stimulus frequency in auditory areas, motor areas (sensorimotor cortex, supplementary motor area) as well as the inferior frontal gyrus and the cerebellum. Tierney and Kraus (2013) demonstrated consistent neural responses in the inferior colliculus (IC) synchronized to a rhythmic auditory stimulus (the syllable “da”). The IC is an early auditory pathway nucleus in the brain stem with rich projections to the cerebellum via the dorsolateral pontine nuclei. Since the cerebellum is activated in sensorimotor synchronization tasks (cf. Stephan et al., 2002; Grahn et al., 2011) and activations in distinct cerebellar regions correspond to different aspects of the temporal dynamics of rhythmic synchronization (Thaut et al., 2009b; Konoike et al., 2012) – such as pattern detection or tracking changes in rhythmic interval duration – the representation of timing information in the IC may be an important function in auditory-to-motor transformations during rhythmic entrainment. Finally, an MEG study by Tecchio et al. (2000) showed amplitude changes in the M100 component of auditory evoked field potential linearly entrained by changes in the rhythmic interval durations, i.e., longer intervals were associated with higher M100 intensities and vice versa. This entrainment pattern was also observable during subliminal duration changes at 2% of the absolute interval duration. However, the exact neural transmission mechanisms from auditory to motor centers have not been fully explored.

Of greatest importance in the context of motor rehabilitation was the finding that the injured brain can indeed access rhythmic entrainment mechanisms. Early studies of gait training in hemiparetic stroke rehabilitation (Thaut et al., 1993, 1997), Parkinson’s disease (Thaut et al., 1996; McIntosh et al., 1997), traumatic brain injury (Hurt et al., 1998), and cerebral palsy (Thaut et al., 1998) confirmed behaviorally the existence of rhythmic entrainment processes in clinical populations. Studies extending entrainment to hemiparetic arm rehabilitation followed closely (Whitall et al., 2000; Thaut et al., 2002).

Rhythmic entrainment established the first testable motor theory for the role of auditory rhythm and music in therapy. The subsequent studies led to the need to codify and standardize rhythmic-musical application for motor rehabilitation (Thaut, 2005; Thaut and Hoemberg, 2014). Theses techniques became the initial foundation of the clinical repertoire of neurologic music therapy.

## TIMING BASED MOVEMENT OPTIMIZATION

The comprehensive effect of rhythmic entrainment on motor control raises some important theoretical questions as to the mechanisms modulating these changes. We know that firing rates of auditory neurons, triggered by auditory rhythm and music, entrain the firing patterns of motor neurons, thus driving the motor system into different frequency levels. There are two additional mechanisms are of great clinical importance in regard to entrainment. The first is that auditory stimulation primes the motor system in a state of readiness to move. Priming increases subsequent response quality.

The second, more specific aspect of entrainment refers to the changes in motor planning and motor execution it creates. Rhythmic stimuli create stable anticipatory time scales or templates. Anticipation is a critical element in improving movement quality. Rhythm provides precise anticipatory time cues for the brain to plan ahead and be ready. Furthermore, successful movement anticipation is based on foreknowledge of the duration of the cue period. During entrainment two movement oscillators – in our case neurally based – of different periods entrain to a common period. In auditory entrainment the motor period entrains to the period of the auditory rhythm. Entrainment is always driven by frequency or period entrainment – that is, the common periods may or may not be in perfect phase lock (i.e., the onset of the motor response would be perfectly synchronized to the auditory beat). Beat entrainment is a commonly misunderstood concept. Entrainment is not defined by beat or phase entrainment – it is defined by period entrainment (Large et al., 2002; Thaut and Kenyon, 2003; Nozaradan et al., 2011).

Period entrainment offers the solution to why auditory rhythm also changes the spatial kinematic and dynamic force measures of muscle activation, e.g., as evidenced by smoothing of velocity and acceleration profiles. Foreknowledge of the duration of the movement period changes computationally everything in motor planning for the brain. Velocity and acceleration are mathematical time derivatives of movement position. Consider that a movement cycle, e.g., of the wrist joint in reaching movements, consists of a finite number of position coordinates *(x,y,z)* each associated with a particular time *(t)* value during the movement period. If we consider, for simplification, the position coordinate *x (t)* to be continuous rather than a discrete function of the following statements can describe mathematically the relationship between position, velocity, and acceleration without going into the mathematical equation detail:

• The velocity *v(t)* at any time *t* is the first time derivative of position *x(t)* and is equal to the numerical value of the slope of the position curve at the time *t*.
• The acceleration *a(t)* at any time *t* is the second time derivative of position *x(t)*, the first time derivative of velocity *v(t)*, and is equal to the numerical value of the slope of the velocity curve at time *t.*
• The position *x* at any time *t* is numerically equal to the area under the velocity-time curve between zero and *t.*
• The velocity v at any time t is numerically equal to the area under the acceleration-time curve between zero and *t*.

Given this background information and using an optimization criterion, e.g., such as minimization of peak acceleration, we can now show that the movement trajectory as a function of time in three-dimensional space is completely determined as a consequence of the optimization condition, i.e., the entire movement cycle is fixed in time due to period entrainment. The fact that an anticipatory temporal constraint on the movement period (given by the stimulus period) results in a kinematically well-defined optimization problem allows for a mathematical analysis showing a complete specification of the three-dimensional coordinates of a limb trajectory. In other words, reduction in trajectory variability of the arm during a reaching movement or the knee during a step cycle is a natural outcome of the rhythmic time constraint.

In clinical language, by fixating movement time through a rhythmic interval the brain’s internal timekeeper now has an additional externally triggered timekeeper with a precise reference interval, a continuous time reference (CTR). This time period presents time information to the brain at any stage of the movement. The brain knows at any point of the movement how much time has elapsed and how much time is left, enabling enhanced anticipatory mapping and scaling of optimal velocity and acceleration parameters across the movement interval. The brain tries to optimize the movement now by matching it to the given template. This process will result not only in changes in movement speed but also in smoother and less variable movement trajectories and muscle recruitment. One can conclude that auditory rhythm, via physiological period entrainment of the motor system, acts as a forcing function to optimize all aspects of motor control. Rhythm not only influences movement timing – time as the central coordinative unit of motor control – but also modulates patterns of muscle activation and control of movement in space (Thaut et al., 1999). Rhythmic cues provide comprehensive optimization information to the brain for re-programming movement.

With this understanding of the underlying mechanisms of entrainment it is clinically less important if the patients synchronize their motor response exactly to the beat – it is important that they entrain to the rhythmic period because the period template contains the critical information to optimize motor planning and motor execution. Research has indeed shown that the timing of the motor response relative to the beat can fluctuate whereas the movement period entrains very quickly and precisely to the rhythmic period and the period entrainment is maintained during frequency changes in the rhythmic stimulus interval (Thaut et al., 1998b).

## CLINICAL APPLICATIONS OF ENTRAINMENT

The insights from rhythmic auditory-motor studies led to a complete re-conceptualization of the role of complex auditory stimuli such as music for therapy and rehabilitation. Traditionally, the role of music in therapy had been considered from social science models as a stimulus for personal interpretation in regard to well-being, emotional response, and social relationship. Although these properties of music are also important for therapeutic functions the new discoveries re-focused the role of music as therapeutic stimulus on its structural properties shaping sensory perception linked to motor function (de l’Etoile, 2010; Altenmueller and Schlaug, 2013).

The early clinical findings have been replicated and extended by a number of other research groups substantiating the existence of rhythmic auditory-motor circuitry for entrainment in hemiparetic gait rehabilitation (Ford et al., 2007; Roerdink et al., 2007, 2011; Thaut et al., 2007; Spaulding et al., 2013). A large number of RAS-Studies have replicated and extended the beneficial use of RAS for mobility in Parkinson’s disease (see for review: deDreu et al., 2012).

After successful experiments entraining endogenous biological rhythms of neural gait oscillators a new question emerged. Can rhythmic entrainment also be applied to entrain whole body movements, especially arm and hand movements that are not driven by underlying biological rhythms? The answer was found by turning functional upper extremity movements, which are usually discrete and non-rhythmic, into repetitive cyclical movement units which now could be matched to rhythmic time cues. Several clinical research studies have successfully investigated rhythmic cuing for upper extremity for full body coordination, especially in hemiparetic stroke rehabilitation (Luft et al., 2004; McCombe-Waller et al., 2006; Schneider et al., 2007; Altenmueller et al., 2009; Malcolm et al., 2009; Grau-Sanchez et al., 2013) and in children with cerebral palsy (Peng et al., 2010; Wang et al., 2013).

The improvements in stroke arm rehabilitation were comparable in size to data from research in constraint induced therapy (CIT; Massie et al., 2009).

## MORE CLINICAL APPLICATIONS OF ENTRAINMENT

Rhythmic entrainment extends beyond motor control. Emerging research shows that speech rate control affecting intelligibility, oral motor control, articulation, voice quality, and respiratory strength may greatly benefit from rhythmic entrainment using rhythm and music (Pilon et al., 1998; Wambaugh and Martinez, 2000; Thaut et al., 2001; Natke et al., 2003; Lim et al., 2013). Recent findings in aphasia rehabilitation suggest that the rhythmic component in melodic intonation therapy may be equally as important as the activation of intact right hemispheric speech circuitry through singing (Stahl et al., 2011).

Lastly, the potential of temporal entrainment of cognitive function has only recently emerged as an important driver of therapeutic change. The recognition that timing and sequencing also have a critical function in cognitive abilities (Conway et al., 2009) has led to research investigating the potential role of music and rhythm as cognitive rehabilitation technique. Sound in music is inherently temporal and sequential and may serve as a “scaffold” to bootstrap the representation of temporal sequential patterns in cognitive functions such as memory (Conway et al., 2009). Bootstrapping non-musical information to the rhythmic-melodic patterns of a musical “scaffold” may offer several advantages to enhance deep encoding during acquisition and retrieval in memory training. Music may cue the temporal order and sequencing of information. The rhythmic-melodic contour may create a pattern structure unto which information units can be mapped. The phrase structure of music patterns may segment the total information units into a smaller set of large chunks or overarching units thus reducing memory load (Wallace, 1994). This last point may constitute a particular advantage in music since musical mnemonics, such as short songs, are usually composed by a small alphabet of pitches and rhythmic motifs (Snyder, 2000). Large information units constructed of large alphabets (e.g., word lists, number tables) can be mapped on a small pitch and rhythm alphabet which is organized into redundant, repetitive, and anticipatory “memory units” reducing memory load and increasing deep encoding (Thaut et al., 2009a).

## FROM ENTRAINMENT TO COMPLEXITY

Neurologic music therapy techniques in cognitive and speech/language rehabilitation are relying to a large extent on the role of timing in music and rhythm. However, the discovery that a musical element such as rhythm can be a very effective driver of therapeutic learning and training, has led to a new look to consider the therapeutic potential of all musical elements within a framework of music perception and music playing. In other words, rhythmic entrainment opened the doors to shifting from predominantly interpretative models of music in therapy to perceptual based models. Interpretative applications of music in therapy remain important, especially when psychosocial, affective/expressive, or associative long-term memory goals become a functional focus of therapy. However, research has now shown how the whole complexity of musical elements can be shaped into functional therapy. Perceptual exercises built on melodic and harmonic patterns in music may be applied to train sustained, selective, divided, focused, and alternating attention in clinical settings (Gardiner and Thaut, 2014). Music as a complex auditory language has been applied to re-training auditory perception deficits and enhance speech perception (Tierney and Kraus, 2013; Mertel, 2014). Special applications of music-based auditory perception training have been used with cochlear-implant users (Mertel, 2014). In patients with hemi-spatial visual neglect therapeutic exercises using music listening and instrument playing that emphasize auditory and visual focus on the neglect side have been shown to be successful in reducing neglect (Hommel et al., 1990; Abiru et al., 2007; Soto et al., 2009; Bodak et al., 2014). Finally, guided elementary composition and improvisation exercises in music emphasizing complexity thinking, decision making, problem solving, reasoning, affective evaluation, self-organization, comprehension, etc. have been shown to be successful in improving executive function in persons with traumatic brain injury (Thaut et al., 2009b; Hegde, 2014).

## FRONTIERS FOR NEUROLOGIC MUSIC THERAPY

The discovery of entrainment for therapeutic purposes in the early 1990s has led to a strong body of research evidence that the periodicity of auditory rhythmic patterns could improve movement patterns in patients with movement disorders. Motor control theory and motor neurophysiology propose that changes in motor patterns are due to priming of the motor system and anticipatory rhythmic templates in the brain that allow for optimal anticipation, motor planning, and execution with an external rhythmic cue. The ability for the brain to use entrainment to re-program the execution of a motor pattern has made rhythmic entrainment an important tool in motor rehabilitation (Thaut and Abiru, 2010; Thaut and McIntosh, 2014). More recently, temporal rhythmic entrainment has been extended into applications in cognitive rehabilitation and speech and language rehabilitation, with emerging evidence that mechanisms of rhythmic entrainment may be an essential tool for rehabilitation in all domains of neurologic music therapy (Thaut, 2010; Thaut and Hoemberg, 2014). The temporal structure of music remains a central element in therapy and rehabilitation. However, the discovery of rhythmic entrainment has also opened the door to exploring the therapeutic mechanisms in other elements of music such as melody and harmony, and finally in the pattern structure of music as a complex auditory language to stimulate and (re)-train complex cognitive functions. Neurologic music therapy as a codified and standardized treatment model, currently comprising 20 techniques in motor, speech/language, and cognitive rehabilitation, has emerged and has been medically been accepted quite rapidly over the past 15 years. However, since NMT was built on existing research data, the future shape of NMT will be dynamically driven by continued research. One of the largest areas of therapeutic need is in psychiatric rehabilitation. Emerging views on the nature of mental illness, driven by new insights from neuropsychiatric research, may allow a more focused extension of NMT techniques in the areas executive and psychosocial function, attention, and memory to contribute to psychiatric treatment.

## Conflict of Interest Statement

The authors declare that the research was conducted in the absence of any commercial or financial relationships that could be construed as a potential conflict of interest.

## References

[B1] Abiru M. Mihara Y. Kikuchi Y. (2007). The effects of neurologic music therapy on hemispatial neglect in a hemiparetic stroke patient: a case study. *Neurol. Med.* 67 88–94.

[B2] Altenmueller E. Marco-Pallares J. Muente T. F. Schneider S. (2009). Neural reorganization underlies improvement in stroke-induced motor dysfunction by music-supported therapy. *Ann. N. Y. Acad. Sci.* 1169 395–405 10.1111/j.1749-6632.2009.04580.x 19673814

[B3] Altenmueller E. Schlaug G. (2013). Neurobiological aspects of neurologic music therapy. *Music Med.* 5 210–216 10.1177/1943862113505328 PMC4425943 25725918

[B4] Bodak R. Malhotra P. Bernardi N. F. Cocchini G. Stewart L. (2014). Reducing chronic visuo-spatial neglect following right hemisphere stroke through instrument playing. *Front. Hum. Neurosci.* 8:413 10.3389/fnhum.2014.00413 PMC4052745 24966827

[B5] Conway C. M. Pisoni D. B. Kronenberger W. G. (2009). The importance of sound for cognitive sequending abilities. *Curr. Dir. Psychol. Sci.* 18 275–279 10.1111/j.1467-8721.2009.01651.x 20725604 PMC2923391

[B6] deDreu M. J. van der Wilk A. S. Poppe E. Kwakkel G. van Wegen E. E. (2012). Rehabilitation, exercise therapy and music in patients with Parkinson’s disease: a meta-analysis of the effects of music-based movement therapy on walking ability, balance, and quality of life. *Parkinsonism Relat. Disord.* 18 114–119 10.1016/S1353-8020(11)70036-0 22166406

[B7] de l’Etoile S. (2010). Neurologic music therapy: a scientific paradigm for clinical practice. *Music Med.* 2 78–84 10.1177/1943862110364232

[B8] Felix R. A. Fridberger A. Leijon S. Berrebi A. S. Magnusson A. K. (2011). Sound rhythms are encoded by postinhibitory rebound spiking in the superior paraolivary nucleus. *J. Neurosci.* 31 12566–12578 10.1523/JNEUROSCI.2450-11.2011 21880918 PMC3712520

[B9] Ford M. Wagenaar R. Newell K. (2007). The effects of auditory rhythms and instruction on walking patterns in individuals post stroke. *Gait Posture* 26 150–155 10.1016/j.gaitpost.2006.08.007 16996270

[B10] Fujioka T. Trainor L. J. Large E. W. Ross B. (2012). Internalized timing of isochronous sounds is represented in neuromagnetic beta oscillations. *J. Neurosci.* 32 1791–1802 10.1523/JNEUROSCI.4107-11.2012 22302818 PMC6703342

[B11] Gardiner J. C. Thaut M. H. (2014). “Musical executive function training,” in“ *Oxford Handbook of Neurologic Music Therapy*, eds Thaut M. H. Hoemberg V. (Oxford: Oxford University Press), 279–293.

[B12] Grahn J. A. Henry M. J. McAuley J. G. (2011). FMRI investigation of crossmodal interactions in beat perception: audition primes vision but not vice versa. *Neuroimage* 54 1231–1243 10.1016/j.neuroimage.2010.09.033 20858544 PMC3002396

[B13] Grau-Sanchez J. Armengual J. L. Rojo N. Vecian de Las heras M. Rubio F. Altenmueller E. (2013). Plasticity in the sensorimotor cortex induced by music-supported therapy in stroke patients: a TMS-study. *Front. Hum. Neurosci.* 7:494 10.3389/fnhum.2013.00494 PMC3759754 24027507

[B14] Hegde S. (2014). Music-based cognitive remediation therapy for patients with traumatic brain injury. *Front. Neurol.* 5:34 10.3389/fneur.2014.00034 PMC3970008 24715887

[B15] Hommel M. Peres B. Pollak P. Memin B. Besson G. Gaio J. M. (1990). Effects of passive tactile and auditory stimuli on left visual neglect. *Arch. Neurol.* 47 573–576 10.1001/archneur.1990.00530050097018 2334306

[B16] Hurt C. P. Rice R. R. McIntosh G. C. Thaut M. H. (1998). Rhythmic auditory stimulation in gait training for patients with traumatic brain injury. *J. Music Ther.* 35 228–241 10.1093/jmt/35.4.228 10519837

[B17] Konoike N. Kotozaki Y. Miyachi S. Miyauchi C. M. Yomogida Y. Akimoto Y. (2012). Rhythm information represented in the fronto-parieto-cerebellar motor system. *Neuroimage* 63 328–338 10.1016/j.neuroimage.2012.07.002 22796994

[B18] Koziol L. F. Budding D. E. (2009). *Suncortical Structures and Cognition: Implications for Neuropsychological Assessment*. New York: Springer 10.1007/978-0-387-84868-6

[B19] Kugler P. N. Turvey M. T. (1987). *Information, Natural Law, and the Self-Assembly of Rhythmic Movement.* Hillside, NJ: Lawrence Erlbaum Assoc. Inc.

[B20] Large E. W. Jones M. R. Kelso J. A. S. (2002). Tracking simple and complex sequences. *Psychol. Res.* 66 3–17 10.1007/s004260100069 11963276

[B21] Lim K. Kim Y. Lee H. Yoo J. Hwang J. Kim J. (2013). The therapeutic effect of neurologic music therapy and speech language therapy in post-stroke aphasic patients. *Ann. Rehabil. Med.* 37 556–562 10.5535/arm.2013.37.4.556 24020037 PMC3764351

[B22] Luft A. R. McCombe-Waller S. Whitall J. (2004). Repetitive bilateral arm training and motor cortex activation in chronic stroke: a randomized controlled trial. *JAMA* 292 1853–1861 10.1001/jama.292.15.1853 15494583 PMC2930817

[B23] Malcolm M. P. Massie C. Thaut M. H. (2009). Rhythmic auditory-motor entrainment improves hemiparetic arm kinematics during reaching movements. *Top. Stroke Rehabil.* 16 69–79 10.1310/tsr1601-69 19443349

[B24] Massie C. Malcolm M. Greene D. Thaut M. H. (2009). Effects of constraint-induced therapy on kinematic outcomes and compensatory movement patterns: an exploratory study. *Arch. Phys. Med. Rehabil.* 90 571–579 10.1016/j.apmr.2008.09.574 19345771

[B25] McCombe-Waller S. Harris-Love M. Liu W. Whitall J. (2006). Temporal coordination of the arms during bilateral simultaneous and sequential movements in patients with chronic hemiparesis. *Exp. Brain Res.* 168 450–454 10.1007/s00221-005-0235-3 16331507

[B26] McIntosh G. C. Brown S. H. Rice R. R. Thaut M. H. (1997). Rhythmic auditory-motor facilitation of gait patterns in patients with Parkinson’s disease. *J. Neurol. Neurosurg. Psychiatry* 62 122–126 10.1136/jnnp.62.1.22 PMC486690 9010395

[B27] Mertel K. (2014). “Auditory perception training,” in *Oxford Handbook of Neurologic Music Therapy*, eds Thaut M. H. Hoemberg V. (Oxford: Oxford University Press), 227–256.

[B28] Moore B. C. J. (2003). *Psychology of Hearing.* New York: Elsevier.

[B29] Natke U. Donath T. M. Kalveram K. T. (2003). Control of voice fundamental frequency in speaking versus singing. *J. Acoust. Soc. Am.* 113 1587–1593 10.1121/1.1543928 12656393

[B30] Nozaradan S. Peretz I. Missal M. Mouraux A. (2011). Tagging the neuronal entrainment to beat and meter. *J. Neurosci.* 31 10234–10240 10.1523/JNEUROSCI.0411-11.2011 21753000 PMC6623069

[B31] Paltsev Y. I. Elner A. M. (1967). Change in functional state of the segmental apparatus of the spinal cord under the influence of sound stimuli and its role in voluntary movement. *Biophysics* 12 1219–1226.

[B32] Pantaleone J. (2002). Synchronization of metronomes. *Am. J. Phys.* 70 992–1000 10.1119/1.1501118

[B33] Peng Y. Lu T. Wang T. Chen Y. Liao H. Lin K. (2010). Immediate effects of therapeutic music on loaded sit-to-stand movement in children with spastic diplegia. *Gait Posture* 33 274–278 10.1016/j.gaitpost.2010.11.020 21185725

[B34] Pilon M. McIntosh K. W. Thaut M. H. (1998). Auditory versus visual timing cues as external rate control to enhance verbal intelligibility in mixed spastic-ataxia dysarthric speakers: a pilot study. *Brain Inj.* 12 793–803 10.1080/026990598122188 9755370

[B35] Roerdink M. Bank P. J. M. Peper C. Beek P. J. (2011). Walking to the beat of different drums: practical implications for the use of acoustic rhythms in gait rehabilitation. *Gait Posture* 33 690–694 10.1016/j.gaitpost.2011.03.001 21454077

[B36] Roerdink M. Lamoth C. J. C. Kwakkel G. van Wieringen P. C. W. Beek P. J. (2007). Gait coordination after stroke: benefits of acoustically paced treadmill walking. *Phys. Ther.* 87 1009–1022 10.2522/ptj.20050394 17553922

[B37] Rossignol S. Melvill Jones G. (1976). Audiospinal influences in man studied by the H-reflex and its possible role in rhythmic movement synchronized to sound. *Electroencephalogr. Clin. Neurophysiol.* 41 83–92 10.1016/0013-4694(76)90217-0 58771

[B38] Schmahmann J. D. Pandya D. N. (2009). *Fiber Pathways of the Brain.* Oxford: Oxford University Press.

[B39] Schneider S. Schoenle P. W. Altenmueller E. Muente T. (2007). Using musical instruments to improve motor skill recovery following stroke. *J. Neurol.* 254 1339–1346 10.1007/s00415-006-0523-2 17260171

[B40] Shelton J. Kumar G. P. (2010). Comparison between auditory and visual single reaction time. *Neurosci. Med.* 1 30–32 10.4236/nm.2010.11004

[B41] Snyder B. (2000). *Music and Memory.* Cambridge, MA: MIT Press.

[B42] Soto D. Funes M. J. Guzmán-García A. Warbrick T. Rotshtein P. Humphreys G. W. (2009). Pleasant music overcomes the loss of awareness in patients with visual neglect. *Proc. Natl. Acad. Sci. U.S.A.* 106 6011–6016 10.1073/pnas.0811681106 19307566 PMC2667081

[B43] Spaulding J. Barber B. Colby M. Cormack B. Mick T. Jenkins M. E. (2013). Cueing and gait improvement among people with Parkinson’s disease: a meta-analysis. *Arch. Phys. Med. Rehabil.* 94 562–570 10.1016/j.apmr.2012.10.026 23127307

[B44] Stahl B. Kotz S. A. Henseler I. Turner R. Geyer S. (2011). Rhythm in disguise: why singing may not hold the key to recovery from aphasia. *Brain* 134 3083–3093 10.1093/brain/awr240 21948939 PMC3187543

[B45] Stephan K. M. Thaut M. H. Wunderlich G. Schicks W. Tian B. Tellmann L. (2002). Conscious and subconscious sensorimotor synchronization: prefrontal cortex and the influence of awareness. *Neuroimage* 15 345–352 10.1006/nimg.2001.0929 11798270

[B46] Tecchio F. Salustri C. Thaut M. H. Pasqualetti P. Rossini P. M. (2000). Conscious vs unconscious adaptation: a MEG study of cerebral responses to rhythmic auditory stimuli. *Exp. Brain Res.* 135 222–220 10.1007/s002210000507 11131507

[B47] Thaut M. H. (2005). *Rhythm, Music, and the Brain: Scientific Foundations and Clinical Applications.* New York: Routledge.

[B48] Thaut M. H. (2010). Neurologic music therapy in cognitive rehabilitation. *Music Percept.* 27 281–285 10.1525/mp.2010.27.4.281

[B49] Thaut M. H. Abiru M. (2010). Rhythmic auditory stimulation in rehabilitation of movement disorders: a review of current research. *Music Percept.* 27 263–269 10.1525/mp.2010.27.4.263

[B50] Thaut M. H. Bin T. Azimi-Sadjadi M. (1998a). Rhythmic finger-tapping sequences to cosine-wave modulated metronome sequences. *Hum. Mov. Sci.* 17 839–863 10.1016/S0167-9457(98)00031-1

[B51] Thaut M. H. Miller R. A. Schauer L. M. (1998b). Multiple synchronization strategies in rhythmic sensorimotor tasks: phase vs. period adaptation. *Biol. Cybern.* 79 241–250 10.1007/s004220050474 9810681

[B52] Thaut M. H. Gardiner J. C. Holmberg D. Horwitz J. Kent L. Andrews G. (2009a). Neurologic music therapy improves executive function and emotional adjustment in traumatic brain injury rehabilitation. *Ann. N. Y. Acad. Sci.* 1169 406–416 10.1111/j.1749-6632.2009.04585.x 19673815

[B53] Thaut M. H. Stephan K. M. Wunderlich G. Schicks W. Tellmann L. Herzog H. (2009b). Distinct cortico-cerebellar activations in rhythmic auditory motor synchronization. *Cortex* 45 44–53 10.1016/j.cortex.2007.09.009 19081087

[B54] Thaut M. H. Hoemberg V. (2014). *Oxford Handbook of Neurologic Music Therapy.* Oxford: Oxford University Press.

[B55] Thaut M. H. Hurt C. P. Dragan D. McIntosh G. C. (1998). Rhythmic entrainment of gait patterns in children with cerebral palsy. *Dev. Med. Child Neurol* 40 15.

[B56] Thaut M. H. Kenyon G. P. (2003). Rapid motor adaptations to subliminal frequency shifts in syncopated rhythmic sensorimotor synchronization. *Hum. Mov. Sci.* 22 321–338 10.1016/S0167-9457(03)00048-4 12967761

[B57] Thaut M. H. Kenyon G. P. Hurt C. P. McIntosh G. C. Hoemberg V. (2002). Kinematic optimization of spatiotemporal patterns in paretic arm training with stroke patients. *Neuropsychologia* 40 1073–1081 10.1016/S0028-3932(01)00141-5 11900758

[B58] Thaut M. H. Kenyon G. P. Schauer M. L. McIntosh G. C. (1999). The connection between rhythmicity and brain function. *IEEE Eng. Med. Biol.* 18 101–108 10.1109/51.752991 10101675

[B59] Thaut M. H. Leins A. Rice R. R. Kenyon G. P. Argstatter H. Fetter M. (2007). Rhythmic auditory stimulation improves gait more than NDT/Bobath training in near ambulatory patients early post stroke: a single-blind randomized control trial. *Neurorehabil. Neural Repair* 21 455–459 10.1177/1545968307300523 17426347

[B60] Thaut M. H. McIntosh G. C. (2014). Neurologic music therapy in stroke rehabilitation. *Curr. Phys. Med. Rehabil. Rep.* 2 106–113 10.1007/s40141-014-0049-y

[B61] Thaut M. H. McIntosh G. C. McIntosh K. W. Hoemberg V. (2001). Auditory rhythmicity enhances movement and speech motor control in patients with Parkinson’s disease. *Funct. Neurol.* 16 163–172. 11495422

[B62] Thaut M. H. McIntosh G. C. Prassas S. G. Rice R. R. (1993). The effect of auditory rhythmic cuing on temporal stride and EMG patterns in hemiparetic gait of stroke patients. *Neurorehabil. Neural Repair* 7 9–16 10.1177/136140969300700103

[B63] Thaut M. H. McIntosh G. C. Rice R. R. (1997). Rhythmic facilitation of gait training in hemiparetic stroke rehabilitation. *J. Neurol. Sci.* 151 207–212 10.1016/S0022-510X(97)00146-9 9349677

[B64] Thaut M. H. McIntosh G. C. Rice R. R. Miller R. A. Rathbun J. Brault J. M. (1996). Rhythmic auditory stimulation in gait training with Parkinson’s disease patients. *Mov. Disord.* 11 193–200 10.1002/mds.870110213 8684391

[B65] Tierney A. Kraus N. (2013). The ability to move to a beat is linked to the consistency of neural responses to sound. *J. Neurosci.* 33 14981–14988 10.1523/JNEUROSCI.0612-13.2013 24048827 PMC6618407

[B66] Wallace W. T. (1994). Memory for music – effect of melody on recall of text. *J. Exp. Psychol. Learn. Mem. Cogn.* 20 1471–1485 10.1037/0278-7393.20.6.1471

[B67] Wambaugh J. L. Martinez A. L. (2000). Effects of rate and rhythm control treatment on consonant production accuracy in apraxia of speech. *Aphasiology* 14 851–871 10.1080/026870300412232

[B68] Wang T. H. Peng Y. C. Chen Y. L. Lu T. W. Liao H. F. Tang P. F. (2013). A home-based program using patterned sensory enhancement improves resistance exercise effects for children with cerebral palsy: a randomized controlled trial. *Neurorehabil. Neural Repair* 10.1177/11545968313491001 23757295

[B69] Whitall J. McCombe Waller S. Silver K. H. Macko R. F. (2000). Repetitive bilateral arm training with rhythmic auditory cuing improves motor function in chronic hemiparetic stroke. *Stroke 31*, 2390–2395 10.1161/01.STR.31.10.2390 11022069

